# A bis-chelate *o*-vanillin-2-ethano­lamine copper(II) complex bearing both imine and amine forms of the ligand

**DOI:** 10.1107/S205698902101166X

**Published:** 2021-11-09

**Authors:** Nataliya Plyuta, Vladimir N. Kokozay, Julia A. Rusanova, Halyna Buvailo, Evgeny Goreshnik, Svitlana Petrusenko

**Affiliations:** a Department of Inorganic Chemistry, Taras Shevchenko National University of Kyiv, Volodymyrska str. 64/13, 01601 Kyiv, Ukraine; b Department of Inorganic Chemistry and Technology, Jožef Stefan Institute, Jamova, cesta 39, SI-1000 Ljubljana, Slovenia

**Keywords:** crystal structure, copper, geometry index, hydrogen bonding

## Abstract

The mol­ecular bis-chelate complex (2-{[(2-hy­droxy­ethyl-κ*O*)amino-κ*N*]meth­yl}-6-meth­oxy­phenolato-κ*O*)(2-{[(2-hy­droxy­eth­yl)imino-κ*N*]meth­yl}-6-meth­oxy­phenolato-κ*O*)copper(II), [Cu(C_10_H_14_NO_3_)(C_10_H_12_NO_3_)] or [Cu(H*L* ^im^)(H*L* ^am^); H*L* ^im^ = C_10_H_14_NO_3_; H*L* ^am^ = C_10_H_12_NO_3_, represents the first compound containing a salicyl­idene-2-ethano­lamine type ligand in both imino H*L* ^im^ (Schiff base) and amino H*L* ^am^ (reduced Schiff base) forms that has been structurally characterized on the basis of X-ray data. Two mol­ecules of the monodeprotonated ligands coordinate the Cu^II^ ion in an (*N*,*O*_phen_)-bidentate and an (*N*,*O*_phen_,*O*_alc_)-tridentate fashion in the case of the imino and amino forms, respectively. The shape of the CuN_2_O_3_ coordination polyhedron is a distorted square-pyramid (geometry index τ_5_ = 0.26). Inter­molecular N—H⋯O and O—H⋯O hydrogen bonds, involving H atoms of the amino and hy­droxy­ethyl groups, create a two-dimensional supra­molecular array extending parallel to (010).

## Abstract 2

The copper(II) atom in the mol­ecular title complex has a distorted square-pyramidal coordination environment by three O and two N atoms from a bidentate and a tridentate ligand.

## Chemical context

Over the last decade, research on transition-metal complexes with salicyl­idene-type Schiff bases (SB) gained a new impetus after a number of highly effective and simple *M*-(SB) catalysts were obtained, where *M* = Cu, Co, Al, *etc* (Payne *et al.*, 2020 ▸; Mitra *et al.*, 2015 ▸; Fei *et al.*, 2014 ▸; Saha *et al.*, 2013 ▸). It has been shown that incorporation of partially or fully reduced Schiff bases (RSB) into the coordination spheres of metal cations can significantly increase their catalytic activities (Liu *et al.*, 2020 ▸; Huo *et al.*, 2021 ▸; Adão *et al.*, 2014 ▸; Sreenivasulu *et al.*, 2005 ▸). Despite the fact that complexes with RSB ligands are supposed to be very promising objects for the creation of new catalysts, information about their syntheses and structures is rather limited. Continuing our work on the elaboration of alternative methods for the synthesis of coordination compounds (Kokozay *et al.*, 2018 ▸), we have investigated the following system: zinc (powder) – copper (powder) – H_2_ *L* – ammonium thio­cyanate – methanol, to prepare heterometallic Cu/Zn complexes with the Schiff base H_2_ *L* ^im^, which is formed *in situ* upon condensation of *o*-vanillin and 2-amino­ethanol. The complex [Cu(H*L* ^im^)(HL^am^)] (where H_2_ *L* ^im^ = 2-[(2-hy­droxy­eth­yl)imino­meth­yl]-6-meth­oxy­phenol; H_2_ *L* ^am^ = 2-[(2-hy­droxy­eth­yl)amino­meth­yl]-6-meth­oxy­phenol) was formed in the reaction mixture as an unintended by-product for which only a few crystals suitable for X-ray analysis were isolated. 
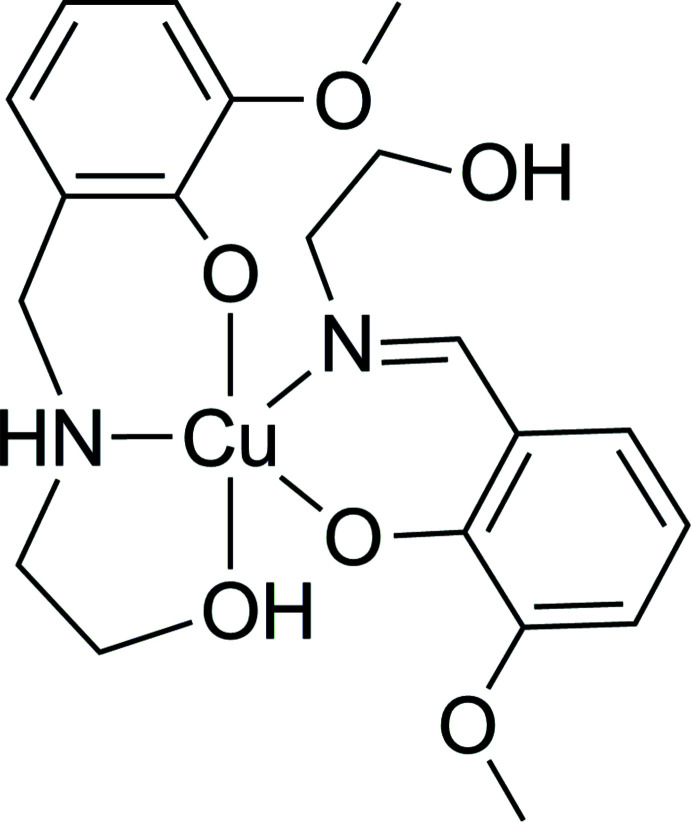


Herein, we report the crystal structure of the title compound, [Cu(H*L* ^im^)(H*L* ^am^)] (I), which represents the first example of a mixed (SB/RSB) complex derived from salicyl­idene-2-amino­ethanol type ligands.

## Structural commentary

**Figure 1:**
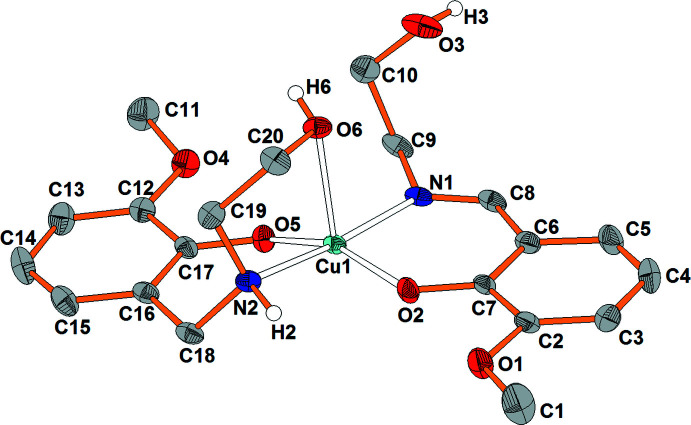
Mol­ecular structure of (I), with the numbering scheme and displacement ellipsoids drawn at the 50% probability level (carbon-bound H atoms are omitted for clarity).

The asymmetric unit of (I) comprises one neutral mol­ecular complex [Cu(H*L* ^im^)(H*L* ^am^)] (Fig. 1 ▸). The copper(II) ion has an O_3_N_2_ coordination set defined by two monodeprotonated mol­ecules of the organic ligands realizing their bidentate (*N,O*) and tridentate (*O,N,O*) functions for the SB and RSB forms, respectively. This difference in coordination behavior of the ligands can be explained by a higher flexibility of the amine ligand, and is observed in similar bis-chelate copper(II) complexes with salicyl­idene-2-amino­ethanol type ligands. Usually, [Cu(SB)_2_] complexes are square-planar and [Cu(RSB)_2_] complexes are octa­hedral. For the corresponding imine complexes, see: Li *et al.* (2005 ▸); Zabierowski *et al.* (2013 ▸, 2014 ▸); Xin *et al.* (2019 ▸); for amine complexes, see: Xie *et al.* (2000 ▸). It is worth noting that such a dependence was not found for similar Ni^II^ complexes, which have an octa­hedral shape *via* both tridentate imino and amino ligands. For [Ni(SB)_2_], see: Floyd *et al.* (2005 ▸); Wang *et al.* (2011*a* ▸,*b* ▸); for [Ni(RSB)_2_], see: Zhang *et al.* (2007 ▸). The shape of the coord­ination polyhedron of the Cu^II^ ion in (I) can be described as distorted [4 + 1] square-pyramidal. The equatorial Cu—O(N) bond lengths vary from 1.923 (2) to 2.030 (3) Å and are in accordance with those found in related complexes (Stetsiuk *et al.*, 2018 ▸; Xie *et al.*, 2000 ▸; Zabierowski *et al.*, 2013 ▸). The length of the long apical Cu—O bond of 2.432 (3) Å lies within the range of Cu^II^—O bond lengths extending up to *ca* 2.70 Å (Alvarez, 2013 ▸). The deviations in *cis* and *trans* [O—Cu—O(N)] angles [80.08 (10)–108.36 (10)° and 157.96 (12)–173.44 (11)°, respectively] are caused by the steric hindrances that are typical for chelate rings. According to the *τ* criterion for five-coordinate complexes (Addison *et al.*, 1984 ▸; O’Sullivan *et al.*, 1999 ▸), the distortion of the CuN_2_O_3_ coordination polyhedron is about 26% along the pathway from regular square-pyramidal to regular trigonal–bipyramidal. The bond-valence sums calculated for Cu^II^ with CN = 4 (1.86 valence units) and CN = 5 (1.99 valence units) (Allmann, 1975 ▸; Shields *et al.*, 2000 ▸) can serve as an additional argument in favor of the coordination number of 5 for Cu^II^ in (I).

## Supra­molecular features

**Figure 2:**
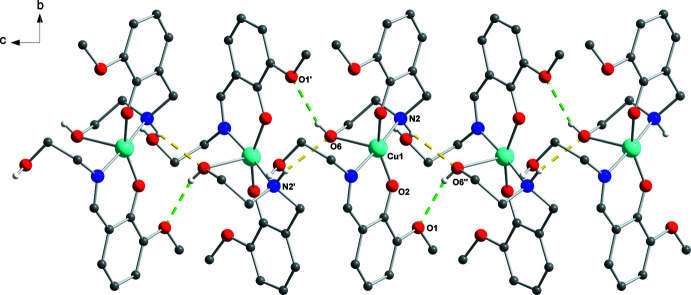
Fragment of the crystal packing of (I), showing inter­molecular O6—H6⋯O1 (green) and N2—H2⋯O6 (yellow) hydrogen bonds forming chains along [001]. [Symmetry codes: (′) 1 − *x*, 1 − *y*, 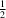 + *z*; (′′) 1 − *x*, 1 − *y*, − 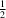 + *z*].

**Figure 3:**
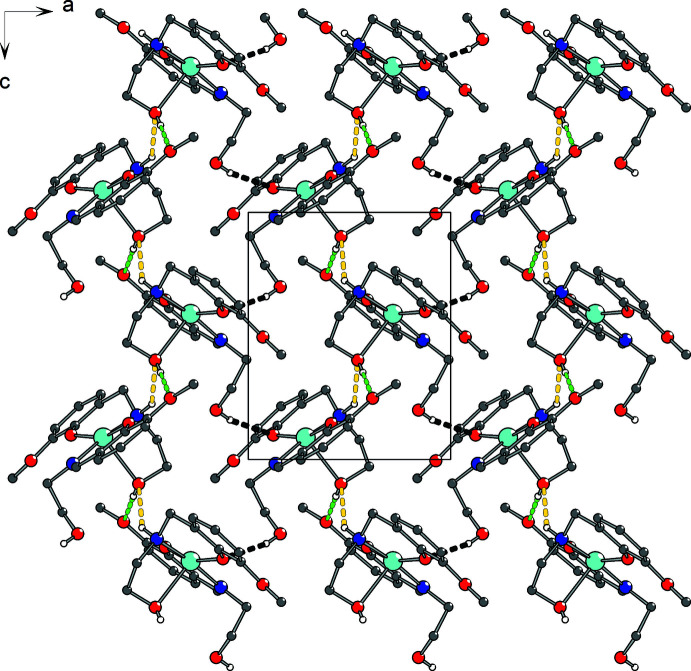
The hydrogen-bonded sheet extending parallel to (010) in the crystal structure of (I). N—H⋯O (yellow) and O—H⋯O hydrogen bonds through participation of coordinating (green) and free (black) hy­droxy­ethyl groups are shown.

Each mol­ecule of (I) forms six inter­molecular hydrogen bonds with four adjacent mol­ecules whereby the following groups take part: non-coordinating hy­droxy­ethyl and amino groups (as H-atom donors), half of the phenolato and meth­oxy groups (as H-atom acceptors) and the coordinating hy­droxy­ethyl groups (both as H-atom donors and acceptors). Chains based on two hydrogen bonds O6—H6⋯O1^ii^ and ^iii^O6⋯H2—N2 (Table 1 ▸, Fig. 2 ▸) are formed along [001]. These chains are linked by O3—H3⋯O5^i^ bonds (Table 1 ▸) into supra­molecular sheets extending parallel to (010) (Fig. 3 ▸).

## Database survey

Among the 33 deposited crystal structures of bis-complexes with a salicyl­idene-2-amino­ethanol-type ligand (CSD, version 5.42, last update February 2021; Groom *et al.*, 2016 ▸), there are 30 hits for complexes with SBs and three hits for complexes with RSBs (Xie *et al.*, 2000 ▸, 2003 ▸; Zhang *et al.*, 2007 ▸). *M*(SB)(RSB) complexes including both forms of a ligand are not known up to now.

## Synthesis and crystallization

*o*-Vanillin (0.3 g, 0.002 mol) and 2-amino­ethanol (0.12 ml, 0.002 mol) were dissolved in methanol and then stirred magnetically at 323–333 K for 20 mins. Copper powder (0.06 g, 0.001 mol), zinc powder (0.07 g, 0.001 mol) and NH_4_SCN (0.15 g, 0.002 mol) were added to the hot yellow solution with further stirring until total dissolution of powder was observed (about 4 h). The resulting brown solution was filtered and left for 1 d. A green powdery precipitate with a few green crystals available for X-ray crystallographic analysis was collected by filtration.

## Refinement

Crystal data, data collection and structure refinement details are summarized in Table 2 ▸. Carbon-bound H atoms were placed in idealized positions and refined using a riding model. H atoms of the NH and OH groups were located in a difference-Fourier map. For the final model they were also treated as riding on their parent atoms.

## Supplementary Material

Crystal structure: contains datablock(s) I. DOI: 10.1107/S205698902101166X/wm5622sup1.cif

Structure factors: contains datablock(s) I. DOI: 10.1107/S205698902101166X/wm5622Isup2.hkl

CCDC reference: 2120197

Additional supporting information:  crystallographic information; 3D view; checkCIF report

## Figures and Tables

**Table 1:** Hydrogen-bond geometry (Å, °)

*D*—H⋯*A*	*D*—H	H⋯*A*	*D*⋯*A*	*D*—H⋯*A*
O3—H3⋯O5^i^	0.82	1.98	2.766 (3)	161
O6—H6⋯O1^ii^	0.75 (5)	2.19 (5)	2.916 (4)	163 (5)
N2—H2⋯O6^iii^	0.98	2.27	3.107 (4)	142

Symmetry codes: (i) -x+2, -y+1, z+{\script{1\over 2}}; (ii) -x+1, -y+1, z+{\script{1\over 2}}; (iii) -x+1, -y+1, z-{\script{1\over 2}}.

**Table 2:** Experimental details

Crystal data	
Chemical formula	[Cu(C_10_H_14_NO_3_)(C_10_H_12_NO_3_)]
*M*_r_	453.97
Crystal system, space group	Orthorhombic, *P* *n* *a*2_1_
Temperature (K)	150
*a*, *b*, *c* (Å)	8.3068 (9), 24.3280 (19), 10.1370 (9)
*V* (Å^3^)	2048.6 (3)
*Z*	4
Radiation type	Mo *K*α
μ (mm^−1^)	1.11
Crystal size (mm)	0.31 × 0.15 × 0.05

Data collection	
Diffractometer	New Gemini, Dual, Cu at zero, Atlas
Absorption correction	Analytical (*CrysAlis PRO*; Rigaku OD, 2015 ▸)
*T*_min_, *T*_max_	0.509, 0.855
No. of measured, independent and observed [*I* > 2σ(*I*)] reflections	10546, 3777, 3539
*R*_int_	0.036
(sin θ/λ)_max_ (Å^−1^)	0.679

Refinement	
*R*[*F* ^2^ > 2σ(*F* ^2^)], *wR*(*F* ^2^), *S*	0.032, 0.079, 1.06
No. of reflections	3777
No. of parameters	268
No. of restraints	1
H-atom treatment	H atoms treated by a mixture of independent and constrained refinement
Δρ_max_, Δρ_min_ (e Å^−3^)	0.31, −0.38
Absolute structure	Classical Flack method preferred over Parsons because s.u. lower
Absolute structure parameter	−0.011 (15)

Computer programs: *CrysAlis PRO* (Rigaku OD, 2015 ▸), *SHELXS* (Sheldrick, 2008 ▸), *SHELXL* (Sheldrick, 2015 ▸), *OLEX2* (Dolomanov *et al.*, 2009 ▸) and *publCIF* (Westrip, 2010 ▸).

## supplementary crystallographic information

### Crystal data

**Table:**

[Cu(C_10_H_14_NO_3_)(C_10_H_12_NO_3_)]	*D*_x_ = 1.472 Mg m^−^^3^
*M**_r_* = 453.97	Mo *K*α radiation, λ = 0.71073 Å
Orthorhombic, *P**n**a*2_1_	Cell parameters from 4616 reflections
*a* = 8.3068 (9) Å	θ = 4.1–28.6°
*b* = 24.3280 (19) Å	µ = 1.11 mm^−^^1^
*c* = 10.1370 (9) Å	*T* = 150 K
*V* = 2048.6 (3) Å^3^	Plate, green
*Z* = 4	0.31 × 0.15 × 0.05 mm
*F*(000) = 948	

### Data collection

**Table:**

New Gemini, Dual, Cu at zero, Atlas diffractometer	3777 independent reflections
Radiation source: fine-focus sealed X-ray tube, Enhance (Mo) X-ray Source	3539 reflections with *I* > 2σ(*I*)
Graphite monochromator	*R*_int_ = 0.036
Detector resolution: 10.6426 pixels mm^-1^	θ_max_ = 28.8°, θ_min_ = 3.5°
ω scans	*h* = −10→9
Absorption correction: analytical (CrysAlisPro; Rigaku OD, 2015)	*k* = −31→31
*T*_min_ = 0.509, *T*_max_ = 0.855	*l* = −13→12
10546 measured reflections	

### Refinement

**Table:**

Refinement on *F*^2^	Hydrogen site location: mixed
Least-squares matrix: full	H atoms treated by a mixture of independent and constrained refinement
*R*[*F*^2^ > 2σ(*F*^2^)] = 0.032	*w* = 1/[σ^2^(*F*_o_^2^) + (0.0466*P*)^2^ + 0.2171*P*] where *P* = (*F*_o_^2^ + 2*F*_c_^2^)/3
*wR*(*F*^2^) = 0.079	(Δ/σ)_max_ = 0.002
*S* = 1.06	Δρ_max_ = 0.31 e Å^−^^3^
3777 reflections	Δρ_min_ = −0.38 e Å^−^^3^
268 parameters	Absolute structure: Classical Flack method preferred over Parsons because s.u. lower
1 restraint	Absolute structure parameter: −0.011 (15)

### Special details

**Table:**

Geometry. All esds (except the esd in the dihedral angle between two l.s. planes) are estimated using the full covariance matrix. The cell esds are taken into account individually in the estimation of esds in distances, angles and torsion angles; correlations between esds in cell parameters are only used when they are defined by crystal symmetry. An approximate (isotropic) treatment of cell esds is used for estimating esds involving l.s. planes.
Refinement. 1. Fixed Uiso At 1.2 times of: All C(H) groups, All C(H,H) groups, All N(H) groups At 1.5 times of: All C(H,H,H) groups, All O(H) groups 2.a Ternary CH refined with riding coordinates: N2(H2) 2.b Secondary CH2 refined with riding coordinates: C9(H9A,H9B), C10(H10A,H10B), C18(H18A,H18B), C19(H19A,H19B), C20(H20A,H20B) 2.c Aromatic/amide H refined with riding coordinates: C3(H3A), C4(H4), C5(H5), C8(H8), C13(H13), C14(H14), C15(H15) 2.d Idealised Me refined as rotating group: C1(H1A,H1B,H1C), C11(H11A,H11B,H11C) 2.e Idealised tetrahedral OH refined as rotating group: O3(H3)

### Fractional atomic coordinates and isotropic or equivalent isotropic displacement parameters (Å^2^)

**Table:**

	*x*	*y*	*z*	*U*_iso_*/*U*_eq_	
Cu1	0.71596 (4)	0.50706 (2)	0.41167 (6)	0.01347 (11)	
O1	0.3846 (3)	0.37604 (10)	0.2532 (3)	0.0268 (6)	
O2	0.5898 (3)	0.44465 (9)	0.3556 (2)	0.0194 (5)	
O3	0.8418 (3)	0.47888 (13)	0.8013 (3)	0.0303 (6)	
H3	0.910708	0.461250	0.840828	0.045*	
O4	1.0725 (3)	0.63655 (10)	0.5057 (3)	0.0219 (5)	
O5	0.8755 (2)	0.56419 (8)	0.4016 (3)	0.0176 (5)	
O6	0.5411 (3)	0.52613 (11)	0.5985 (3)	0.0187 (5)	
H6	0.567 (5)	0.5466 (18)	0.649 (5)	0.028*	
N1	0.8651 (3)	0.46065 (12)	0.5178 (3)	0.0154 (6)	
N2	0.5490 (3)	0.55538 (11)	0.3227 (3)	0.0155 (6)	
H2	0.475620	0.530360	0.276506	0.019*	
C1	0.2700 (6)	0.33956 (19)	0.1958 (5)	0.0428 (12)	
H1A	0.200504	0.359757	0.137664	0.064*	
H1B	0.206937	0.322833	0.264295	0.064*	
H1C	0.325208	0.311543	0.146868	0.064*	
C2	0.4986 (4)	0.35356 (15)	0.3365 (3)	0.0196 (7)	
C3	0.5087 (5)	0.29840 (15)	0.3675 (3)	0.0245 (8)	
H3A	0.434851	0.273654	0.332428	0.029*	
C4	0.6303 (5)	0.27994 (15)	0.4515 (4)	0.0303 (9)	
H4	0.636593	0.242899	0.473592	0.036*	
C5	0.7397 (5)	0.31605 (16)	0.5012 (4)	0.0255 (8)	
H5	0.821347	0.303071	0.555711	0.031*	
C6	0.7321 (4)	0.37315 (15)	0.4720 (3)	0.0187 (7)	
C7	0.6087 (4)	0.39289 (12)	0.3878 (3)	0.0155 (7)	
C8	0.8504 (4)	0.40836 (15)	0.5316 (3)	0.0184 (7)	
H8	0.924904	0.391349	0.586611	0.022*	
C9	0.9906 (5)	0.48739 (15)	0.5970 (4)	0.0208 (8)	
H9A	1.048384	0.513686	0.542842	0.025*	
H9B	1.066860	0.460018	0.627594	0.025*	
C10	0.9173 (5)	0.51648 (16)	0.7138 (4)	0.0246 (8)	
H10A	1.000746	0.536379	0.760726	0.029*	
H10B	0.838544	0.542958	0.682967	0.029*	
C11	1.1634 (5)	0.67643 (17)	0.5768 (4)	0.0309 (9)	
H11A	1.234707	0.695184	0.517408	0.046*	
H11B	1.225148	0.658469	0.644258	0.046*	
H11C	1.091562	0.702508	0.616645	0.046*	
C12	0.9635 (3)	0.65617 (12)	0.4146 (4)	0.0180 (6)	
C13	0.9540 (4)	0.71095 (14)	0.3758 (4)	0.0247 (8)	
H13	1.025083	0.736751	0.410207	0.030*	
C14	0.8373 (5)	0.72677 (16)	0.2852 (4)	0.0294 (9)	
H14	0.832475	0.763044	0.256536	0.035*	
C15	0.7285 (5)	0.68872 (16)	0.2376 (4)	0.0249 (8)	
H15	0.648537	0.699987	0.179372	0.030*	
C16	0.7364 (4)	0.63357 (14)	0.2756 (3)	0.0172 (7)	
C17	0.8560 (4)	0.61619 (13)	0.3639 (3)	0.0156 (7)	
C18	0.6167 (4)	0.59254 (15)	0.2197 (3)	0.0191 (7)	
H18A	0.529395	0.612378	0.177709	0.023*	
H18B	0.669693	0.570550	0.152753	0.023*	
C19	0.4499 (4)	0.58501 (13)	0.4204 (4)	0.0201 (7)	
H19A	0.354505	0.599835	0.378298	0.024*	
H19B	0.510825	0.615315	0.457572	0.024*	
C20	0.4019 (4)	0.54520 (15)	0.5289 (4)	0.0220 (8)	
H20A	0.329361	0.563357	0.589924	0.026*	
H20B	0.345529	0.514124	0.490604	0.026*	

### Atomic displacement parameters (Å^2^)

**Table:**

	*U*^11^	*U*^22^	*U*^33^	*U*^12^	*U*^13^	*U*^23^
Cu1	0.01244 (19)	0.01677 (18)	0.01119 (17)	0.00087 (13)	−0.0031 (2)	0.0002 (3)
O1	0.0330 (15)	0.0203 (12)	0.0270 (14)	−0.0059 (11)	−0.0155 (11)	0.0038 (11)
O2	0.0223 (13)	0.0170 (11)	0.0191 (11)	0.0016 (10)	−0.0084 (10)	0.0035 (10)
O3	0.0191 (13)	0.0563 (18)	0.0155 (12)	0.0059 (14)	−0.0001 (11)	0.0035 (13)
O4	0.0167 (12)	0.0237 (12)	0.0254 (12)	−0.0012 (10)	−0.0079 (11)	−0.0016 (11)
O5	0.0133 (10)	0.0172 (9)	0.0224 (11)	0.0013 (8)	−0.0023 (12)	0.0018 (13)
O6	0.0148 (13)	0.0261 (13)	0.0152 (11)	0.0016 (10)	−0.0013 (10)	−0.0025 (11)
N1	0.0091 (13)	0.0265 (15)	0.0107 (12)	0.0014 (11)	−0.0009 (10)	0.0005 (12)
N2	0.0123 (13)	0.0199 (14)	0.0143 (12)	−0.0019 (11)	−0.0028 (11)	−0.0014 (12)
C1	0.047 (3)	0.032 (2)	0.049 (3)	−0.017 (2)	−0.029 (2)	0.006 (2)
C2	0.0239 (19)	0.0221 (17)	0.0126 (15)	0.0020 (14)	−0.0013 (14)	0.0024 (14)
C3	0.030 (2)	0.0193 (16)	0.0239 (17)	−0.0026 (15)	−0.0004 (15)	−0.0018 (15)
C4	0.037 (2)	0.0180 (17)	0.036 (2)	0.0062 (16)	−0.0021 (17)	0.0066 (16)
C5	0.028 (2)	0.0234 (19)	0.0253 (18)	0.0093 (15)	−0.0033 (16)	0.0062 (17)
C6	0.0193 (18)	0.0226 (18)	0.0143 (17)	0.0049 (14)	0.0016 (13)	0.0019 (15)
C7	0.0187 (16)	0.0168 (13)	0.0109 (17)	0.0015 (12)	0.0027 (12)	0.0024 (13)
C8	0.0133 (17)	0.0290 (19)	0.0131 (15)	0.0095 (14)	0.0000 (13)	0.0046 (15)
C9	0.0138 (18)	0.033 (2)	0.0157 (16)	−0.0009 (15)	−0.0048 (15)	0.0074 (16)
C10	0.024 (2)	0.0272 (19)	0.0228 (18)	0.0012 (17)	−0.0099 (17)	−0.0019 (16)
C11	0.029 (2)	0.029 (2)	0.035 (2)	−0.0051 (17)	−0.0109 (18)	−0.0042 (18)
C12	0.0134 (14)	0.0215 (14)	0.0191 (14)	0.0022 (11)	0.0007 (17)	0.000 (2)
C13	0.0205 (18)	0.0205 (15)	0.033 (2)	−0.0040 (13)	0.0023 (15)	0.0013 (15)
C14	0.027 (2)	0.0220 (18)	0.039 (2)	0.0011 (16)	0.0012 (18)	0.0126 (17)
C15	0.0217 (19)	0.027 (2)	0.025 (2)	0.0035 (15)	−0.0027 (15)	0.0091 (17)
C16	0.0152 (17)	0.0231 (17)	0.0132 (16)	0.0018 (13)	0.0022 (13)	0.0021 (15)
C17	0.0144 (16)	0.0184 (15)	0.0139 (14)	0.0030 (13)	0.0054 (12)	0.0038 (13)
C18	0.0179 (18)	0.0265 (18)	0.0129 (15)	0.0031 (14)	−0.0018 (13)	0.0026 (15)
C19	0.0137 (15)	0.0221 (14)	0.0245 (17)	0.0014 (11)	0.0019 (16)	−0.0019 (19)
C20	0.0126 (18)	0.0302 (19)	0.0232 (18)	0.0016 (14)	0.0009 (14)	0.0037 (16)

### Geometric parameters (Å, º)

**Table:**

Cu1—O2	1.930 (2)	C5—C6	1.422 (5)
Cu1—O5	1.923 (2)	C6—C7	1.417 (5)
Cu1—O6	2.432 (3)	C6—C8	1.437 (5)
Cu1—N1	1.992 (3)	C8—H8	0.9300
Cu1—N2	2.030 (3)	C9—H9A	0.9700
O1—C1	1.426 (5)	C9—H9B	0.9700
O1—C2	1.382 (4)	C9—C10	1.508 (5)
O2—C7	1.310 (4)	C10—H10A	0.9700
O3—H3	0.8200	C10—H10B	0.9700
O3—C10	1.420 (5)	C11—H11A	0.9600
O4—C11	1.425 (4)	C11—H11B	0.9600
O4—C12	1.379 (4)	C11—H11C	0.9600
O5—C17	1.332 (4)	C12—C13	1.392 (5)
O6—H6	0.75 (5)	C12—C17	1.417 (5)
O6—C20	1.432 (4)	C13—H13	0.9300
N1—C8	1.286 (5)	C13—C14	1.390 (5)
N1—C9	1.467 (5)	C14—H14	0.9300
N2—H2	0.9800	C14—C15	1.381 (6)
N2—C18	1.491 (4)	C15—H15	0.9300
N2—C19	1.476 (4)	C15—C16	1.397 (5)
C1—H1A	0.9600	C16—C17	1.402 (5)
C1—H1B	0.9600	C16—C18	1.518 (5)
C1—H1C	0.9600	C18—H18A	0.9700
C2—C3	1.381 (5)	C18—H18B	0.9700
C2—C7	1.422 (5)	C19—H19A	0.9700
C3—H3A	0.9300	C19—H19B	0.9700
C3—C4	1.396 (5)	C19—C20	1.519 (5)
C4—H4	0.9300	C20—H20A	0.9700
C4—C5	1.360 (6)	C20—H20B	0.9700
C5—H5	0.9300		
			
O2—Cu1—O6	93.16 (10)	N1—C9—H9A	109.5
O2—Cu1—N1	92.92 (11)	N1—C9—H9B	109.5
O2—Cu1—N2	87.35 (11)	N1—C9—C10	110.5 (3)
O5—Cu1—O2	157.96 (12)	H9A—C9—H9B	108.1
O5—Cu1—O6	108.36 (10)	C10—C9—H9A	109.5
O5—Cu1—N1	90.55 (10)	C10—C9—H9B	109.5
O5—Cu1—N2	91.65 (10)	O3—C10—C9	111.5 (3)
N1—Cu1—O6	93.37 (10)	O3—C10—H10A	109.3
N1—Cu1—N2	173.44 (11)	O3—C10—H10B	109.3
N2—Cu1—O6	80.08 (10)	C9—C10—H10A	109.3
C2—O1—C1	117.4 (3)	C9—C10—H10B	109.3
C7—O2—Cu1	128.1 (2)	H10A—C10—H10B	108.0
C10—O3—H3	109.5	O4—C11—H11A	109.5
C12—O4—C11	116.8 (3)	O4—C11—H11B	109.5
C17—O5—Cu1	128.2 (2)	O4—C11—H11C	109.5
Cu1—O6—H6	119 (3)	H11A—C11—H11B	109.5
C20—O6—Cu1	99.2 (2)	H11A—C11—H11C	109.5
C20—O6—H6	111 (3)	H11B—C11—H11C	109.5
C8—N1—Cu1	124.1 (2)	O4—C12—C13	123.9 (3)
C8—N1—C9	116.5 (3)	O4—C12—C17	114.8 (3)
C9—N1—Cu1	119.1 (2)	C13—C12—C17	121.2 (3)
Cu1—N2—H2	106.1	C12—C13—H13	120.3
C18—N2—Cu1	113.8 (2)	C14—C13—C12	119.5 (3)
C18—N2—H2	106.1	C14—C13—H13	120.3
C19—N2—Cu1	111.4 (2)	C13—C14—H14	119.9
C19—N2—H2	106.1	C15—C14—C13	120.1 (3)
C19—N2—C18	112.6 (3)	C15—C14—H14	119.9
O1—C1—H1A	109.5	C14—C15—H15	119.4
O1—C1—H1B	109.5	C14—C15—C16	121.1 (3)
O1—C1—H1C	109.5	C16—C15—H15	119.4
H1A—C1—H1B	109.5	C15—C16—C17	119.9 (3)
H1A—C1—H1C	109.5	C15—C16—C18	119.8 (3)
H1B—C1—H1C	109.5	C17—C16—C18	120.2 (3)
O1—C2—C7	113.5 (3)	O5—C17—C12	118.1 (3)
C3—C2—O1	124.4 (3)	O5—C17—C16	123.8 (3)
C3—C2—C7	122.1 (3)	C16—C17—C12	118.1 (3)
C2—C3—H3A	120.2	N2—C18—C16	112.7 (3)
C2—C3—C4	119.7 (3)	N2—C18—H18A	109.1
C4—C3—H3A	120.2	N2—C18—H18B	109.1
C3—C4—H4	119.9	C16—C18—H18A	109.1
C5—C4—C3	120.1 (3)	C16—C18—H18B	109.1
C5—C4—H4	119.9	H18A—C18—H18B	107.8
C4—C5—H5	119.2	N2—C19—H19A	109.9
C4—C5—C6	121.6 (4)	N2—C19—H19B	109.9
C6—C5—H5	119.2	N2—C19—C20	108.7 (3)
C5—C6—C8	117.7 (3)	H19A—C19—H19B	108.3
C7—C6—C5	119.2 (3)	C20—C19—H19A	109.9
C7—C6—C8	123.1 (3)	C20—C19—H19B	109.9
O2—C7—C2	118.6 (3)	O6—C20—C19	110.6 (3)
O2—C7—C6	124.2 (3)	O6—C20—H20A	109.5
C6—C7—C2	117.2 (3)	O6—C20—H20B	109.5
N1—C8—C6	127.5 (3)	C19—C20—H20A	109.5
N1—C8—H8	116.2	C19—C20—H20B	109.5
C6—C8—H8	116.2	H20A—C20—H20B	108.1
			
Cu1—O2—C7—C2	178.7 (2)	C5—C6—C7—C2	−0.3 (5)
Cu1—O2—C7—C6	−0.9 (5)	C5—C6—C8—N1	179.7 (3)
Cu1—O5—C17—C12	153.4 (3)	C7—C2—C3—C4	−0.1 (5)
Cu1—O5—C17—C16	−27.4 (5)	C7—C6—C8—N1	−1.2 (6)
Cu1—O6—C20—C19	−44.8 (3)	C8—N1—C9—C10	−102.7 (3)
Cu1—N1—C8—C6	2.6 (5)	C8—C6—C7—O2	0.2 (5)
Cu1—N1—C9—C10	71.2 (3)	C8—C6—C7—C2	−179.4 (3)
Cu1—N2—C18—C16	−62.6 (3)	C9—N1—C8—C6	176.1 (3)
Cu1—N2—C19—C20	−44.5 (3)	C11—O4—C12—C13	9.3 (5)
O1—C2—C3—C4	179.1 (3)	C11—O4—C12—C17	−169.1 (3)
O1—C2—C7—O2	1.8 (4)	C12—C13—C14—C15	2.2 (6)
O1—C2—C7—C6	−178.6 (3)	C13—C12—C17—O5	177.7 (3)
O4—C12—C13—C14	−178.6 (3)	C13—C12—C17—C16	−1.6 (5)
O4—C12—C17—O5	−3.9 (5)	C13—C14—C15—C16	−2.2 (6)
O4—C12—C17—C16	176.9 (3)	C14—C15—C16—C17	0.3 (6)
N1—C9—C10—O3	63.7 (4)	C14—C15—C16—C18	−179.0 (3)
N2—C19—C20—O6	63.9 (4)	C15—C16—C17—O5	−177.6 (3)
C1—O1—C2—C3	0.1 (5)	C15—C16—C17—C12	1.5 (5)
C1—O1—C2—C7	179.4 (4)	C15—C16—C18—N2	−134.9 (3)
C2—C3—C4—C5	−0.9 (6)	C17—C12—C13—C14	−0.3 (6)
C3—C2—C7—O2	−178.9 (3)	C17—C16—C18—N2	45.8 (4)
C3—C2—C7—C6	0.7 (5)	C18—N2—C19—C20	−173.8 (3)
C3—C4—C5—C6	1.3 (6)	C18—C16—C17—O5	1.6 (5)
C4—C5—C6—C7	−0.7 (6)	C18—C16—C17—C12	−179.2 (3)
C4—C5—C6—C8	178.5 (4)	C19—N2—C18—C16	65.4 (3)
C5—C6—C7—O2	179.3 (3)		

### Hydrogen-bond geometry (Å, º)

**Table:**

*D*—H···*A*	*D*—H	H···*A*	*D*···*A*	*D*—H···*A*
O3—H3···O5^i^	0.82	1.98	2.766 (3)	161
O6—H6···O1^ii^	0.75 (5)	2.19 (5)	2.916 (4)	163 (5)
N2—H2···O6^iii^	0.98	2.27	3.107 (4)	142

Symmetry codes: (i) −*x*+2, −*y*+1, *z*+1/2; (ii) −*x*+1, −*y*+1, *z*+1/2; (iii) −*x*+1, −*y*+1, *z*−1/2.
